# A Strategy to Valorize a By-Product of Pine Wood (*Pinus pinaster*) for Copper Removal from Aqueous Solutions

**DOI:** 10.3390/molecules28186436

**Published:** 2023-09-05

**Authors:** Chiara Mongioví, Maélys Jaillet, Dario Lacalamita, Nadia Morin-Crini, Michael Lecourt, Sandra Tapin-Lingua, Grégorio Crini

**Affiliations:** 1 Chrono-Environnement, Université de Franche-Comté, CNRS, Faculté des Sciences, 25000 Besançon, France; chiara.mongiovi@univ-fcomte.fr (C.M.); maelys.mydh@gmail.com (M.J.); dario.lacalamita@univ-fcomte.fr (D.L.); nadia.crini@univ-fcomte.fr (N.M.-C.); 2 Institut FCBA, Institut Technologique Forêt Cellulose Bois-Construction Ameublement, Domaine Universitaire, CS 90251, cedex 9, 38044 Grenoble, France; michael.lecourt@fcba.fr (M.L.); sandra.tapin-lingua@fcba.fr (S.T.-L.)

**Keywords:** wood, copper, adsorption, water decontamination

## Abstract

This study describes the valorization of a pine wood by-product (*Pinus pinaster*) in the form of individualized fibers to a complex copper or more broadly metals present in an aqueous solution using a batch process. The adsorption results show that pine fibres activated by sodium carbonate are effective in recovering copper ions from monocontaminated or polycontaminated solutions of varying concentrations in a few minutes. One gram of material captures 2.5 mg of copper present in 100 mL of solution at pH 5 in less than 10 min. The results are perfectly reproducible and independent of pH between 3 and 5. The presence of the Na^+^ cation at concentrations of 0.1 M has no impact on material performance, unlike that of Ca^2+^ ions, which competes with Cu^2+^ ions for active sites. The adsorption process can be considered as rapid, as most of the copper is adsorbed within the first 10 min of exposure. Investigation of modeling possibilities shows some limitations. Indeed, the Weber and Morris and Elovich models show poor possibilities to describe all the kinetic data for copper adsorption on fibres. This may prove that the mechanism is far more complex than simple physisorption, chemisorption and/or diffusion. Complexation by wood fibers can be extended to solutions containing several types of metals. The results of this study show that the field of selective metal recovery could be a new way of valorizing by-products from the wood industry.

## 1. Introduction

In Europe, under the 2000 European Water Directive (WFD), companies that use large volumes of water are classified for environmental protection (ICPE), and their substance discharges are controlled to comply with regulatory values known as emission limit values (ELVs). ICPE industries have to consider improving the treatment of their discharge in order to improve chemical and ecological quality of water [1,2]. In France, the Région Bourgogne Franche-Comté is particularly concerned with the problem of metal emissions. It is a region of excellence, with a large number of companies in the surface treatment (ST) sector of various industries, from automotive to aeronautics, electricity, metallurgy, tanning and luxury goods (watchmaking, fashion industry, leather goods) [3].

The ST sector uses large volumes of water and chemical substances, and consequently produces significant quantities of wastewater contaminated by a mixture of substances with variable pH levels and high salinity. Among these substances, copper occupies an important place. This metallic element is considered a priority substance by the French Water Agency for ecological water quality. In addition, along with seven other metals (cadmium, mercury, arsenic, lead, nickel, chromium and zinc), copper is used to calculate an index called METOX (article R. 213-48-3 of the French Environment Code) to establish a toxicity threshold linked to the importance of metals present in the aquatic environment. Copper, as a trace element and an industrial substance, is indispensable; it is found in many products (food supplements, for example) and used in many sectors (agriculture, viticulture, livestock farming, pharmaceuticals, cosmetology, etc.). However, it can also be toxic if present in high concentrations. This is why this metal is the subject of special attention [4,5,6,7].

In general, the technique used to decontaminate metal-contaminated water is physico-chemical, which involves precipitating the metal elements by adding an alkalizing agent such as lime or soda and separating the clarified water from the sludge formed by filtration or sedimentation processes [8]. If the discharge complies with current regulations (for copper, the ELV is 2 mg/L), it is released back to the environment. The current context is increasingly focused on preserving the quality of water resources; consequently, manufacturers are being asked to implement techniques capable of achieving zero discharge of pollutants, or at least of significantly reducing the ELV down to 2 mg/L for copper. However, this is not an easy task, since metals are present in trace amounts and are accompanied by the organic load in complex polycontaminated and highly saline mixtures. In theory, there are several techniques available to achieve a higher level of decontamination, complementary to chemical insolubilization, the physico-chemical technique traditionally used by the ST sector to remove metals from their wastewater. Coupling physico-chemistry with complementary treatments such as membrane filtration (nanofiltration, reverse osmosis), adsorption, ion exchange or evaporation makes it possible to achieve the objective of zero pollutant discharge. Each of these methods has its advantages and disadvantages [9].

In recent years, a new challenge has emerged: recovering metals present in wastewater (diluted effluents, concentrated baths) or in treated water (discharges) in trace amounts and recycling them [5,8,9,10,11]. The aim in this case is to recover metals instead of discharging them into the environment. This challenge is relevant considering the current crisis situation (rising raw material prices, financial speculation, shortages, environmentally unfriendly extraction of metals, etc.). One of the techniques capable of retaining and complexing metals is ion exchange. This treatment method is already used by ST manufacturers to produce demineralized water, protect treatment baths and maintain their quality, recycle rinses, or reduce the ELVs of the main metals (Cu, Ni, Cr, Al, etc.) present in water treated by physical chemistry [9]. The ion exchange technology is also mainly used to recover high-value metals (Au, Ag, Pd, Pt) present in low-concentration water. However, the ion exchange technology is highly sensitive to the presence of suspended solids (SS), organic load (characterized by the values of chemical oxygen demand, COD), salts, and pH. This is why, in wastewater treatment plants (WWTPs), ion exchange is coupled with preliminary stages of filtration to remove SS and activated carbon adsorption to remove COD. However, for small- and medium-sized businesses, operating and maintenance costs can be significant. In addition, it is difficult to effectively and selectively remove metals from complex mixtures, especially because of the competition between different chemical species and the specific parameters (highly variable pH, high salinity) of each type of effluent. In addition, carbon reactors and resin filters are known to quickly become saturated. They must then either be replaced (in the case of coals), or regenerated (resins), which generates additional costs. Therefore, effective, simple, viable and environmentally friendly techniques for recovering metals are to be identified [7,9,11,12,13,14,15,16,17,18,19,20,21,22,23,24,25].

The adsorption of pollutants on inexpensive lignocellulosic materials is well known [26,27,28,29,30,31,32,33]. These media are used as biosorbents in ion exchange, chelation or adsorption processes to complex metals, dyes, hydrocarbons, bacteria and viruses present in an aqueous solution or in leachates. Numerous studies have been published, and the materials proposed take the form of sawdust, bark and hurds (shives), for example, from wood, hemp and flax, either raw or chemically modified. Lignocellulosic materials modified by grafting carboxylic functions onto their surface have been proposed as adsorbents because of the ion exchange properties of these functions. This grafting strongly modifies their affinities for dyes or their selectivity towards hard or soft metals, as, for example, in [7,12,14,15,26,27,30,34,35,36,37,38,39,40,41,42,43,44,45]. Fewer studies have been carried out on fibers extracted directly from wood, without chemical modification or grafting [14,15,44].

Maritime pine (*Pinus pinaster*) is a cultivated species in southern Europe. It is used in a variety of applications: as lumber in sawmills for applications in construction, furniture and processing industries such as particleboard or pulp for paper. Controlled mechanical defibration of wood produces isolated fibers or fiber bundles that can be recovered in bulk or in panels by dedicated plants. New ways of adding value to the wood industry by-products need to be identified to strengthen the sector. This study is part of an industrial project to develop low-cost, effective technologies for treating wastewater contaminated with metals such as copper from the surface treatment process. Two industrial partners are heavily involved: The first wishes to explore new ways of adding value to wood, and more specifically maritime pine fibers, and the second wishes to selectively recover the copper present in concentrated baths, finding methods of treating polycontaminated wastewater capable of reducing the copper emission limit value set at 2 mg/L for its discharges. To our knowledge, this is the first time that pine material has been used for these purposes.

In this study, wood fibers, activated by sodium carbonate Na_2_CO_3_, are used as an adsorbent material of complex copper ions present in synthetic solutions reproducing real-life conditions of industrial baths and discharges. The complexation properties of the materials, obtained by the batch method and expressed in terms of adsorption capacity or % reduction, are described. The influence of several batch variables such as initial copper concentration, material dose, contact time, initial solution pH, as well as the presence of salts (NaCl, CaCl_2_) in the solution to be treated is evaluated and discussed. Experimental data are modeled using equations (adsorption isotherms and kinetic models) commonly used in the literature.

## 2. Results and Discussion

**Figure 1:**
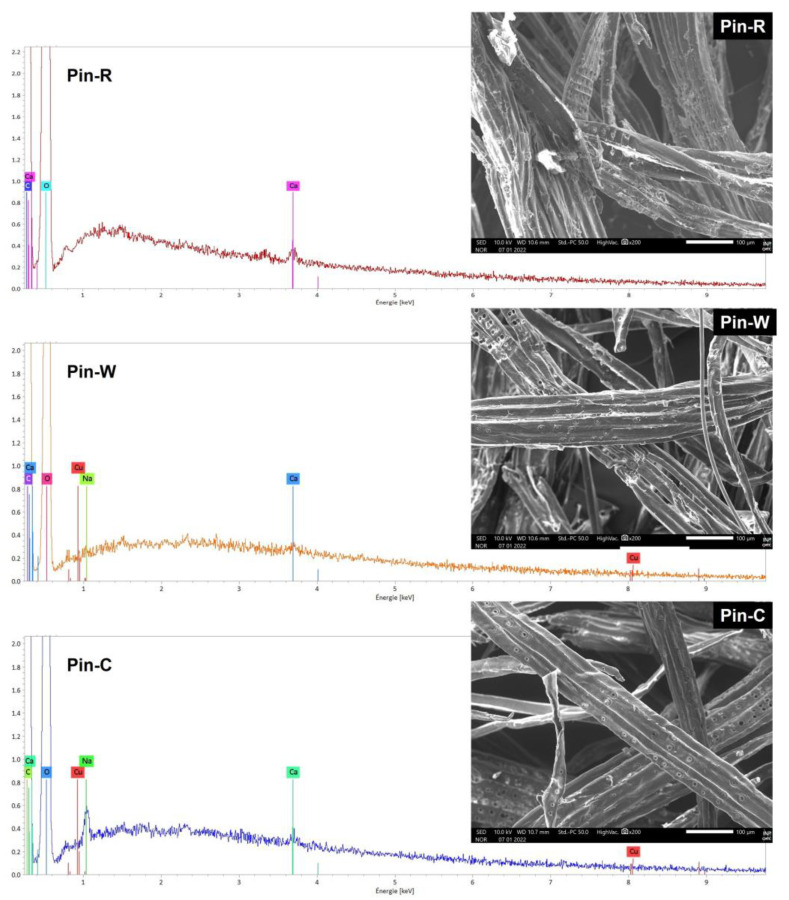
Comparison of scanning electron microscopy images and elemental analysis spectra by energy-dispersive spectroscopy of raw pine (Pin-R), washed pine (Pin-W) and activated pine (Pin-C) materials.

The activation of materials with sodium carbonate is a process proposed by the industrial partner to activate carboxylic functions. Preliminary studies have shown that this type of activation leads to changes in both the structure of materials and their chemical composition, in line with numerous published studies on the subject [13,14,15,44]. SEM images and EDS spectra of maritime pine materials in raw (Pin-R), washed (Pin-W) and sodium carbonate-activated (Pin-C) forms are shown in Figure 1. In the spectrum of the Pin-R sample, the presence of the calcium signal can be observed, which disappears after a simple water wash. For the Na_2_CO_3_-activated sample (Pin-C), a new sodium signal is observed. SEM images also show a smoother, softer surface for Pin-W and Pin-C materials. Some fine elements observed onto the fibers generate during mechanical processing are washed away.

**Figure 2:**
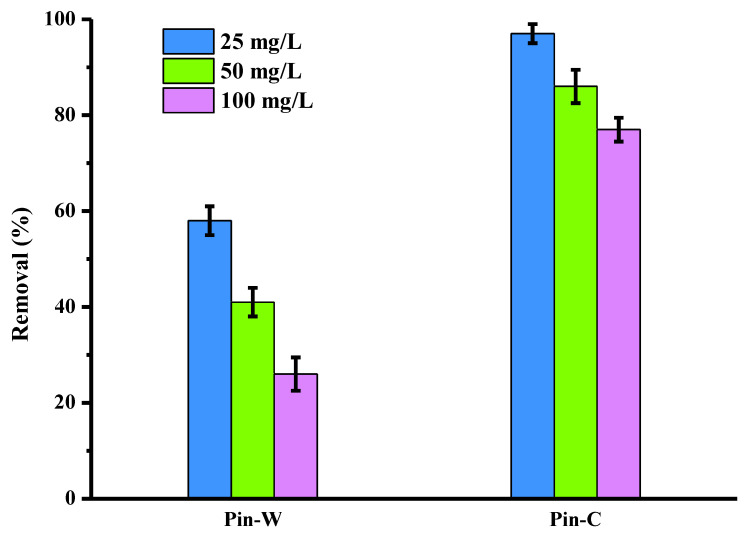
Comparison of copper abatements (expressed in % of removed pollutant) obtained on two pine materials, washed (Pin-W) and activated (Pin-C), at three initial concentrations of copper (other conditions: volume of solution = 100 mL; mass of material = 1 g; contact time = 120 min; T = 22 ± 1 °C; pH_i_ = 5.1–5.4 ± 0.1; agitation = 250 rpm; n = 5).

Figure 2 compares the copper elimination obtained on washed (Pin-W) and activated (Pin-C) pine materials, for three initial copper concentrations present in an aqueous solution with an initial pH close to 5 (pH generally encountered in industrial water from the surface treatment process). The copper elimination is expressed in % of removed pollutant (abatements). Whatever the concentration, the abatements measured for sodium carbonate-activated pine are always higher than those for washed pine. These eliminations are high: 1 g of the activated material is capable of complexing all of the 2.5 g of copper introduced in a 100 mL volume, the reduction being close to 100%, whereas for washed pine, the value is close to 60%. This result demonstrates the interest of activating the adsorbent material with sodium carbonate, as carboxylic acids are converted into carboxylate functions, which then enable the metal cations to be complexed. This interpretation was confirmed by two phenomena: the first concerned the systematic increase in the final pH value, indicating the presence of chemical interactions; the second concerned the increase in sodium concentrations found in the supernatants after adsorption, confirming interactions by ion exchange, with the sodium cation carried by the active sites of the fibers being replaced by the metal cation in the aqueous solution. Similar interpretations have been reported in the literature [13,40,41,44]. Finally, the results in Figure 2 were repeated five times under identical batch conditions, and the standard deviation values showed the reproducibility of the data.

**Figure 3:**
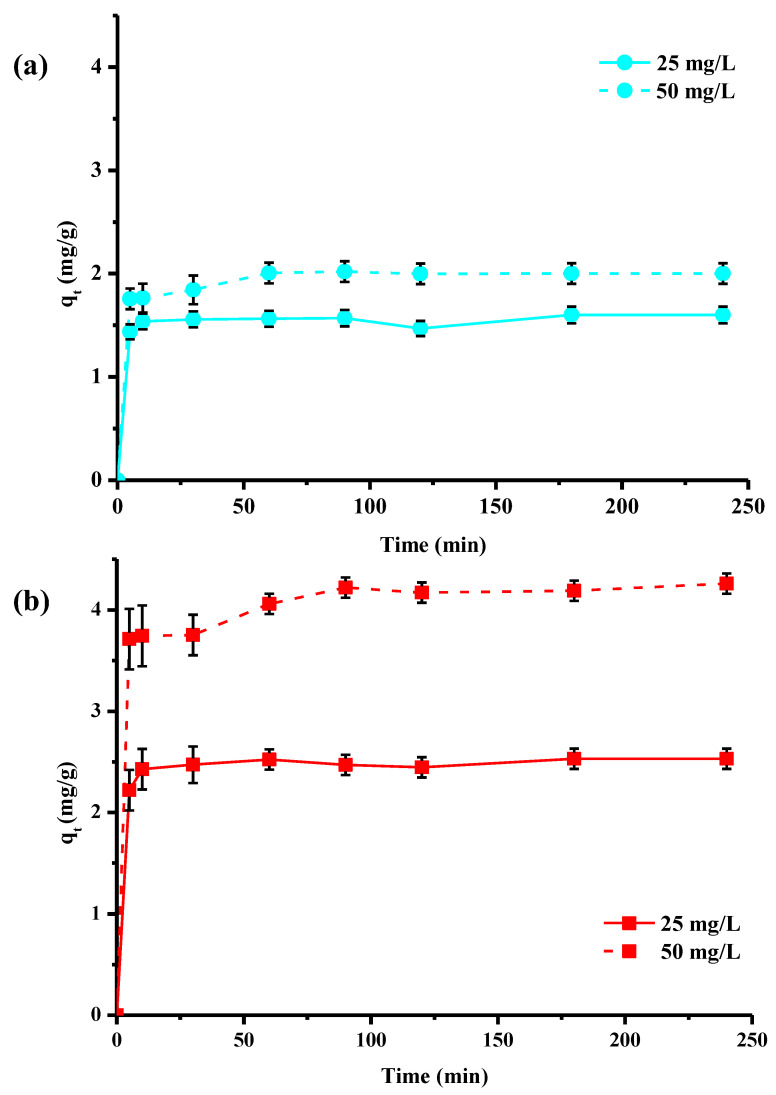
Kinetics of adsorption of copper ions by washed Pin-W (**a**) and activated Pin-C (**b**) pine materials at two different concentrations, 25 and 50 mg/L (other conditions: solution volume = 100 mL; mass of material = 1 g; T = 22 ± 1 °C; pH_i_ = 5.2–5.4 ± 0.1; stirring = 250 rpm; n = 3).

The results in Figure 2 were obtained with a contact time of 120 min, the same as that used by the manufacturers in their batch tests. Nevertheless, the effect of contact time on copper removal by both materials was investigated (Figure 3). The study was conducted over a time range from 5 to 240 min and at two different copper concentrations, 25 and 50 mg/L, with the other batch conditions held constant. The experimental results were represented by plotting the amount of adsorbed copper (q_t_ in mg/g) at time t as a function of the time variable (t in min). The data in Figure 3 show that q_t_ values increase with time and initial copper concentration until equilibrium is reached. Irrespective of concentration, higher adsorbed quantities were obtained for the activated material compared to the washed pine. At the start of the adsorption process, the presence of a large number of accessible open active sites on the surface favored rapid copper adsorption: q_t_ values increased very rapidly. Then, as contact time was extended, ions moved through the pores of the adsorbent (internal diffusion). The number of free sites and their accessibility then began to decrease, and reaction equilibrium was reached. The data clearly indicated that the adsorption process can be considered rapid, since the greatest quantity of copper was adsorbed by the fibers within the first 10 min of adsorption. This result can be explained by the presence of strong interactions between the materials and the metal ions via surface adsorption, diffusion into the wood material network and chemisorption.

**Figure 4:**
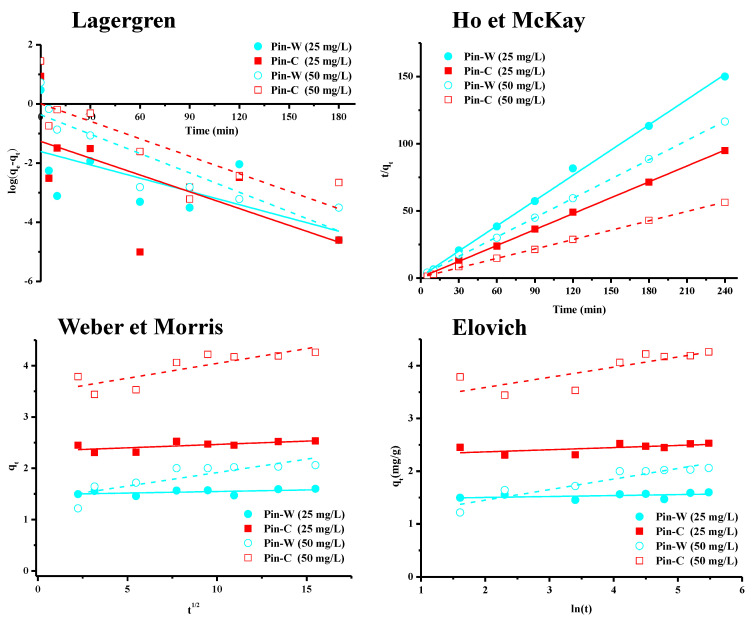
Graphical representations of the kinetic models of Lagergren, Ho and McKay, Weber and Morris, and Elovich for Pin-W washed pine and Pin-C activated pine materials at two different concentrations, 25 and 50 mg/L.

Experimental kinetic data were modeled using various empirical models commonly used in the literature [46,47,48,49,50,51,52,53]. The graphical representations of these models are shown in Figure 4, and the calculated parameters are presented in Table 1. The first model used is the pseudo-first-order model established by Lagergren [54], which considers that the occupancy rate of adsorption sites is proportional to the number of unoccupied sites. This model is expressed by Equation (3), in which q_e_ and q_t_, respectively, represent the quantity of metal adsorbed at equilibrium and time t (mg/g), and k_1_ is the pseudo-first-order rate constant (min^−1^). Interpretation of these results showed that Lagergren’s model did not apply to our data set (Figure 4; Table 1), and that copper was therefore not adsorbed by maritime pine fibers by occupying a localized adsorption site.

The second model is that of Ho and McKay, or the pseudo-second-order model [55], which is widely used in the literature due to its simplicity. It is expressed by Equation (4) in which q_e_ and q_t_ represent, respectively, the amount of metal adsorbed at equilibrium and time t (mg/g) and k_2_ is the pseudo-second-order rate constant (g/mg min). Plotting 1/q_t_ as a function of time t systematically yields a linear plot with regression coefficients close to one (Table 1). The application of the Ho and McKay model enabled us to fit our experimental data to those obtained by calculation (Figure 4, Table 1). Indeed, the q_e_ values found theoretically by this model were closer to those found experimentally than those calculated by the pseudo-first-order model. However, this model, being empirical and without scientific basis, provided no information on the adsorption mechanism. Like the Lagergren model, the Ho and McKay model simply suggested that adsorption depends on the adsorbent–adsorbent couple. In the literature, other simplified models such as those of Weber and Morris (intraparticle diffusion model) [56] or Elovich (chemisorption or ion exchange model) [57] have been used to obtain information on these mechanisms.

The intraparticle diffusion model, widely used in the literature despite numerous recent criticisms, is based on a concept of diffusion in the pores of the material. In addition to the models of Lagergren and Ho and McKay, the applicability of the Weber–Morris model makes it possible to hypothesize the interactions (physisorption, chemisorption and, above all, diffusion) involved in the adsorption process, and thus to define the limit stage of the kinetics [56]. The graphical representation of this model (Equation (5)) offers a constant for the rate of intraparticle diffusion (mg/g min^1/2^) and an index for the thickness of the boundary layer (the higher the index, the thicker the boundary layer). In general, three lines were observed: the first, and clearest, corresponded to the external diffusion stage (film diffusion), the second represented the internal or intraparticle diffusion stage, and the third was the final equilibrium stage (adsorption reaction). Analysis of kinetic data over the whole range from 0 to 240 min contact time showed the non-adjustment of experimental data with those calculated from the Weber and Morris intraparticle diffusion model (Table 1). The values of the correlation coefficients were indeed low for the entire time range studied. Consequently, for Pin-W and Pin-C materials and for both copper concentrations, the data set did not follow the Weber and Morris model, so that internal or external diffusion was not the main mechanism controlling adsorption kinetics.

The final model is that of Elovich [57]. In general, this chemisorption model is used to describe adsorption processes characterized by the presence of a heterogeneous surface and to assess the chemical nature of the adsorption mechanism. However, the R^2^ values obtained for the two pine materials showed that this model did not apply to the experimental data (Table 1), regardless of the concentration used.

The structure of the fibers, made up of tangles of cellulose microfibrils coated with hemicellulose and lignin in the outer layer, and the anatomy of the fibers, with a more or less accessible lumen, justify such an approach to diffusion and chemisorption. However, a full analysis of the modeling via these Weber and Morris and Elovich models has shown that neither of these two models is entirely satisfactory in describing all the kinetic data for copper adsorption on maritime pine. The mechanism is far more complex than simple physisorption, chemisorption and/or diffusion [46,47,48,49,50,51,52,53]. Indeed, lignocellulosic materials such as those in this work are known to have a complex structure, with fibers consisting of tangles of cellulose microfibrils covered with hemicellulose and lignin in the outer layer, as well as fiber anatomy with a more or less accessible lumen.

**Figure 5:**
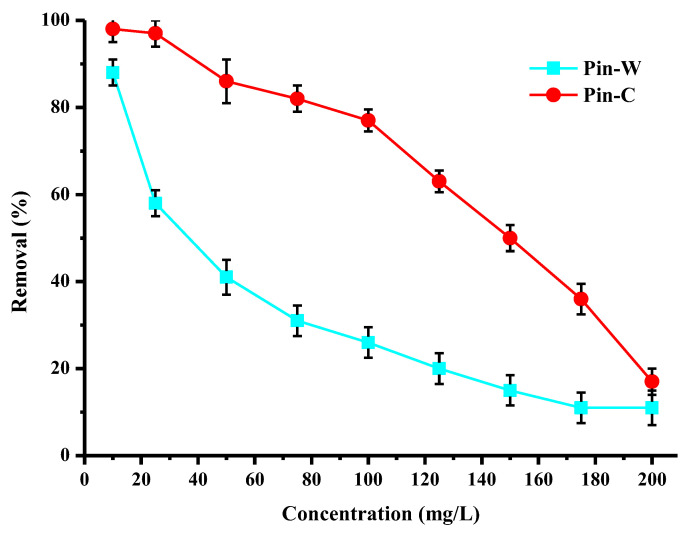
Effect of the initial copper concentration on the purification performance (expressed in % of removed copper) of washed (Pin-W) and activated (Pin-C) pine materials (other conditions: volume of solution = 100 mL; mass of material = 1 g; contact time = 120 min; T = 22 ± 1 °C; pH_i_ = 4.7–5.6 ± 0.1; stirring = 250 rpm; n = 3).

The results in Figure 5 describe the impact of initial copper concentration on the purification performance of washed and activated pine materials. For this study, a series of experiments was carried out with initial concentrations ranging from 10 to 200 mg/L, while all other batch conditions were constant. For the same quantity of wood fiber, increasing the concentration lead to a decrease in the abatement rate through progressive saturation of the active sites. Activated pine once again demonstrated high performance even at high copper concentrations, confirming the interest of the chemical activation of the material. Indeed, for an initial concentration of 100 mg/L, the abatement rate of activated pine was of the order of 80%, and could therefore enable the recovery of copper present in industrial baths. For concentrations of industrial interest (below 10 mg/L), Pin-C was capable of complexing all the copper present in the solution, and thus of achieving the decontamination objective (reduction of ELV < 2 mg/L). Finally, however, it should be noted that the abatement rate observed for washed pine did not follow the same trend as that for activated pine.

To determine the theoretical isotherm followed by copper adsorption by maritime pine fibers and predict, by calculation, the maximum adsorption capacity (q_m_) of this material, three empirical equations were applied: Langmuir [58], Freundlich [59], and Temkin [60]. The simplest model used in the literature to describe adsorption properties is that proposed by Langmuir. This simplified model is given by Equation (7), where q_e_ represents the equilibrium adsorption capacity (mg/g), Ce is the equilibrium concentration (mg/L) and K is the Langmuir constant (L/g). Its graphical representation enables us to estimate the maximum adsorption capacity of a given material (q_m_ in mg/g), corresponding to adsorbent saturation [58]. According to the assumptions of this model, the following information can be deduced: uniform material surface, localized adsorption on a fixed number of sites, instantaneous and reversible reaction, identical active sites capable of binding only one pollutant, no interaction between pollutants, constant adsorption energy and monolayer adsorption with the existence of a saturation plateau value [3,48,50].

The Freundlich equation is also widely used. According to this model, the material surface is heterogeneous and interactions are possible between the adsorbates forming a multilayer [59]. In addition, this empirical equation assumes that there is a large number of active sites available on the material surface, acting simultaneously, each with a different adsorption free energy. Adsorption is reversible and infinite. In Equation (8), K_F_ is the Freundlich constant (mg^1-(1/n)^ L^1/n^/g) and n_F_ is a heterogeneity coefficient. The value of 1/n_F_ is between zero and one and indicates the degree of non-linearity between solution concentration and adsorption. If the value of 1/n_F_ is equal to unity, adsorption is linear; if the value is less than unity, this implies that the adsorption process is favorable; and if the value is greater than unity, the adsorption is an unfavorable process. The more heterogeneous the surface, the closer the value of 1/n_F_ to zero.

Temkin and Pyzhev studied the effects of certain indirect interactions between adsorbate and adsorbate on adsorption isotherms [60]. They suggested that, due to these interactions and ignoring concentration values, the heat of adsorption of all adsorbates in the layer would decrease linearly with coverage. This concept is expressed by the so-called Temkin Equation (9), in which K_T_ is the equilibrium binding constant (L/g) corresponding to the maximum binding energy and B is another constant (J/mol) related to the adsorption energy [46,49].

**Figure 6:**
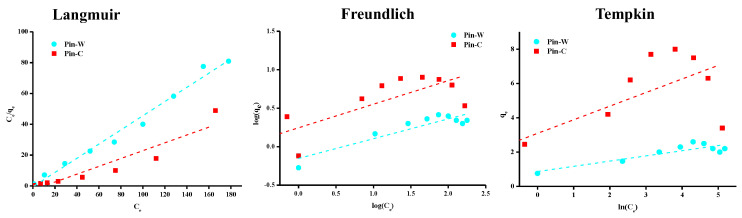
Representations of adsorption isotherms of experimental results modelled by the Langmuir, Freundlich and Temkin equations.

The results described in Figure 6 and Table 2, respectively, show graphical representations of the experimental data modeled by these three equations and the corresponding parameters calculated. Interpretation of these data showed a better match with the experimental data by applying the Langmuir model. The Langmuir equation can also be used to calculate q_m_: for Pin-W and Pin-C, values of 2.2 and 3.9 mg of copper per gram of material, respectively, were obtained. Similar values were recently reported in the literature [7]. These values are of the same order of magnitude as those described in the literature for other lignocellulosic materials. The data in Table 2 show 1/n_F_ values below one, indicating a favorable adsorption process. Finally, the Temkin and Pyzhev model is not applicable to our data set.

**Figure 7:**
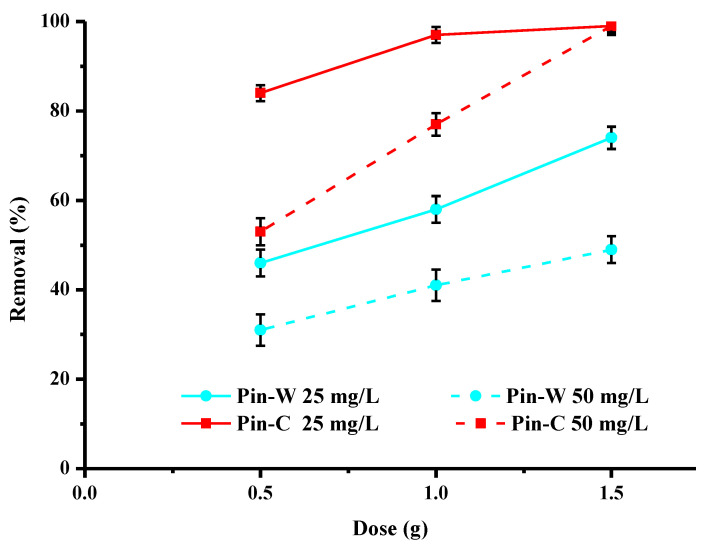
Effect of the mass of adsorbent used on copper abatement (expressed in % of removed pollutant) by washed (Pin-W) and activated (Pin-C) pine materials at two different concentrations, 25 and 50 mg/L (other conditions: volume of solution = 100 mL; contact time = 120 min; T = 22 ± 1 °C; pH_i_ = 5.2–5.4 ± 0.1; agitation = 250 rpm; n = 3).

The data in Figure 7 describe the effect of the initial dose of washed and activated materials on the adsorption of copper present at concentrations of 25 and 50 mg/L. These results show that, for the same initial concentration, the increase in the concentration of maritime pine fibres in the water leads to an increase in the abatement rate due to the increase in the number of active sites available for the adsorption of metal cations. For activated pine, a dose of 1 g is sufficient to obtain the highest purifying percentages at the concentration of 25 mg/L. For 50 mg/L of copper, abatement rates are lower. However, to increase these levels, it is enough to increase the amount of fiber present in the water to be treated. The replacement of a conventional adsorbent by a bioadsorbent implies that the latter has a similar or even better performance at the same or lower cost, which is the case, our material being an abundant and cheap co-product.

**Figure 8:**
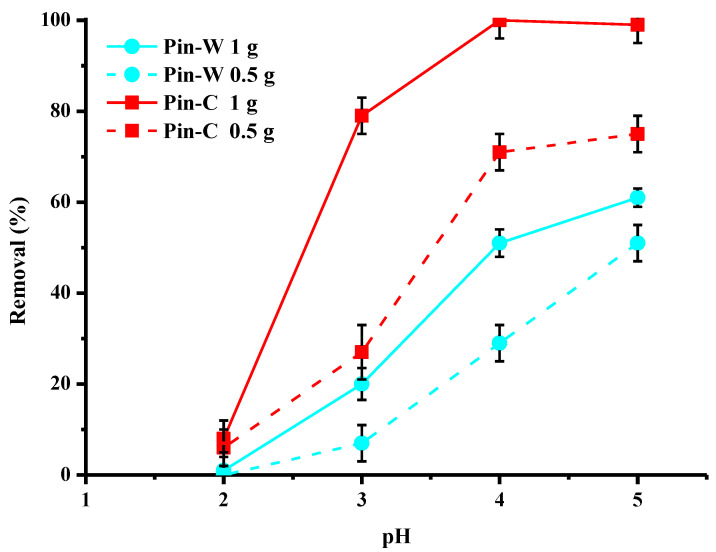
Effect of the initial pH of the solution on copper abatement (expressed in % of removed pollutant) by washed (Pin-W) and activated (Pin-C) pine materials at two different masses, 0.5 and 1 g (other conditions: volume of solution = 100 mL; [Cu] = 25 mg/L; contact time = 120 min; T = 22 ± 1 °C; stirring = 250 rpm; n = 3).

One of the most important parameters in an adsorption process is pH because it modifies both the surface charge of the adsorbents and the chemistry of the elements to be complexed. Figure 8 describes the effect of the initial pH of the solution to be treated on the reduction in copper by the two types of fibers at two different doses of copper. This study was carried out for pH values between two and five because at values above five, copper begins to precipitate [7]. Analysis of the data showed that the optimal pH range for the highest performance is between four and five, especially for activated pine. At the two doses studied, the trends were identical for both materials. The lower the pH, the lower the abatement values. Especially at pH 2, copper abatement was very low, because at this pH, cupric ions compete with H+ ions for active sites [7].

**Figure 9:**
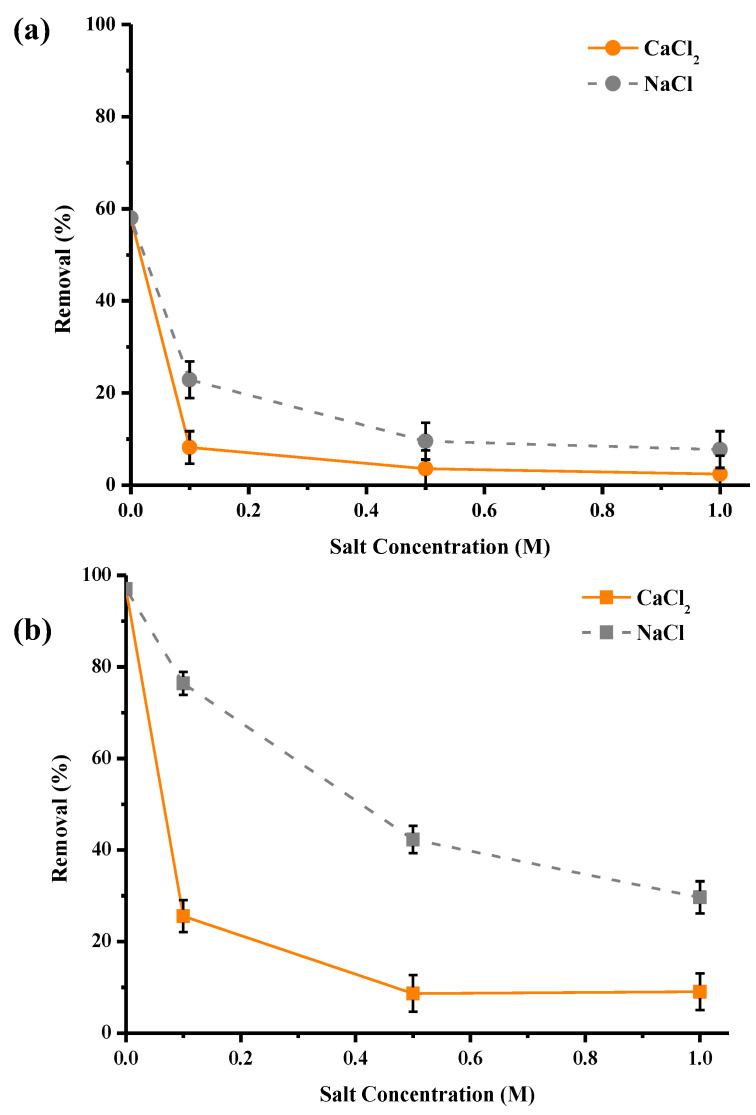
Effect of presence of NaCl and CaCl_2_ in the solution on copper abatement (expressed in % of removed pollutant) by Pin-W (**a**) and activated Pin-C (**b**) pine materials (other conditions: volume of solution = 100 mL; mass of material = 1 g; [Cu] = 25 mg/L; [Na^+^] = [Ca^2+^] = 0.1, 0.5 and 1 mol/L; contact time = 120 min; T = 22 ± 1 °C; stirring = 250 rpm; pH = 5.4 ± 0.1; n = 3).

In order to study the impact of ionic strength on the purification performance of materials, different concentrations of NaCl and CaCl_2_ salts were explored, as these salts are widely used in surface treatment. Three molar concentrations were studied: 0.1, 0.5 and 1 mol/L, which correspond to 5.8, 29 and 58 g/L for NaCl and 11, 55 and 110 g/L for CaCl_2_. Values between 0.1 and 0.5 M are representative of those found in effluents and industrial discharges. The results in Figure 9 show a significant drop in the purification performance of materials with the increase in salt concentration. Indeed, the adsorption rates for washed pine decreased from 58% to 10% for Na+ and from 58% to 5% for Ca^2+^, respectively, in the absence of salt and at a concentration of 1 M of Na^+^ or Ca^2+^. For activated pine, the abatements were reduced from 97% to 30% for Na^+^ and 9% for Ca^2+^. These results can be explained by the suppression of electrostatic interactions. Indeed, when the amount of salt increased, i.e., an increase in the ionic strength of the solution occurred, the amount of Ca^2+^ or Na^+^ ions increased, which masked the active sites of pine fibers for Cu^2+^ ions. Nevertheless, at the concentration of 25 mg/L of copper and in the case of the addition of NaCl, activated pine was able to remove about 30% of copper even at the concentration of 1 M of Na^+^, which demonstrates a very important selectivity. Another interesting observation was that the presence of the Ca^2+^ cation had more effect on copper adsorption than the presence of the Na^+^ cation, which can be explained by the fact that Ca^2+^ ions are more similar to Cu^2+^ ions in terms of their physical and chemical properties (ionic radius, charge, etc.). There is, therefore, competition between Ca^2+^ and Cu^2+^ ions for active sites.

**Figure 10:**
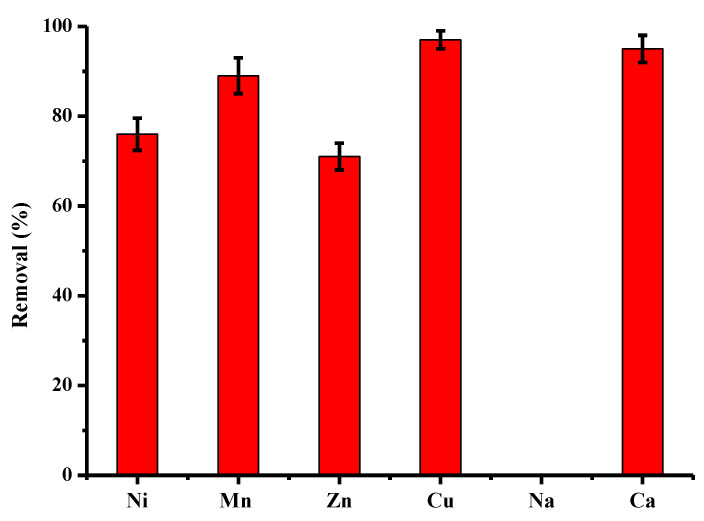
Comparison between adsorption capacities (expressed in % of removed pollutant) of activated pine material towards several cations present in monocontaminated solution at a concentration of 25 m/L (other conditions: volume of solution = 100 mL; mass of material = 1 g; contact time = 120 min; T = 22 ± 1 °C; stirring = 250 rpm; pH_i_ = 5.4–5.9 ± 0.1; n = 5).

Some industrial baths resulting from the processes are generally characterized by high concentrations of copper (of the order of g/L) but relatively insignificantly contaminated by other chemical species. On the contrary, in industrial discharges that are sent into aquatic environments, copper can be found, but also other cations such as Ni, Mn, Zn, Na and Ca. However, the concentrations are lower, of the order of a few tens of mg/L. The results of Figure 10 describe the purification performance of activated pine material towards several cations present in a monocontaminated solution at a concentration of 25 mg/L. Other batch conditions are constant, as in the case of monocontaminated copper solutions. For this concentration, the following order of affinity was obtained: Cu > Ca > Mn > Ni ~ Zn >>> Na, with abatements of 100%, 96%, 90%, 76%, 71% and 0%. The sodium was not adsorbed at all by the treated maritime pine and was even released into solution in all experiments, which confirms the exchange of ions. On the contrary, calcium was largely adsorbed by activated pine, which showed a great affinity of this material vis-à-vis the mineral. The difference in the degree of biosorption can be attributed to the physical and chemical characteristics of each element, namely ionic radius, molar mass and electronegativity [61].

**Figure 11:**
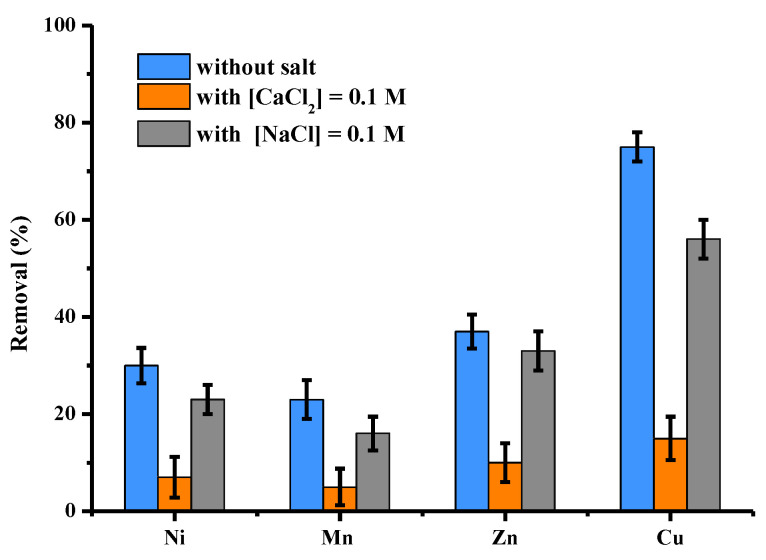
Comparison between adsorption capacities (expressed in % of removed pollutant) of activated pine material towards 4 cations (Ni, Mn, Zn and Cu) present in polycontaminated solution at a total concentration of 100 mg/L in the presence or without CaCl_2_ and NaCl (other conditions: volume of solution = 100 mL; mass of material = 1 g; individual concentration of each cation = 25 mg/L; [Na^+^] = [Ca^2+^] = 0.1 mol/L contact time = 120 min; T = 22 ± 1 °C; stirring = 250 rpm; pH = 5.3 ± 0.1; n = 3).

Adsorption experiments in solutions polycontaminated with four metals (Ni, Mn, Zn and Cu) were performed by selecting the initial concentration of each metal of 25 mg/L or 100 mg/L in total metals. The influence of the presence of Na^+^ and Ca^2+^ ions was studied by adding to the initial solutions a quantity of salt at the concentration of 0.1 mol/L, i.e., 2.3 g of Na^+^ and 4 g of Ca^2+^. The results are shown in Figure 11. In the mixture, regardless of the experience, the order of affinity was as follows: Cu >> Zn > Ni > Mn. This order was independent of the presence of salt. Of the 10 mg of metals present in the 100 mL of the initial solution, 1 g of activated pine adsorbed 4.12 mg of the four metals. In the presence of NaCl or CaCl_2_, this value increased to three, 21 and 0.92 mg, respectively. Activated pine, therefore, has an excellent biosorption capacity for the four metals present in the polycontaminated solution, in particular for the 2.5 mg copper present in the 100 mL of the batch solution. In addition, it can be seen that this selectivity for copper remains important despite the presence of Na^+^ and Ca^2+^ ions.

**Figure 12:**
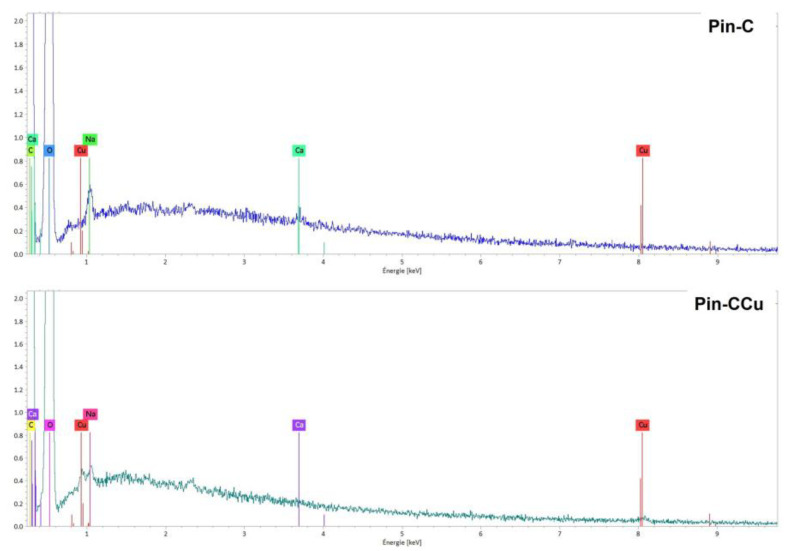
EDS spectra of Na_2_CO_3_ activated maritime pine samples before (Pin-C) and after adsorption (Pin-CCu).

Characterization analyses by EDS spectroscopy were performed for the fibers of Pin-C after copper adsorption (Pin-CCu). On the spectrum obtained after adsorption, a new peak due to copper could be observed (Figure 12).

## 3. Materials and Methods

### 3.1. Materials

Maritime pine fibers derived from a mechanical defibration process were supplied by the FCBA Institute (France). These fibers were treated by simple washing with water and activation with sodium carbonate Na_2_CO_3_, according to a protocol developed by the industrial partner. Table 3 describes the chemical composition of the washed and carbonate-treated fibers. Extractive content was measured using the acetone-water extraction sequence on a Soxtec Automatic extractor apparatus. Klason lignin content was performed according to Tappi T-222 om-02 standard methods.

### 3.2. Synthetic Solutions

The studied metals were copper, zinc, manganese and nickel, the cationic species found in ST effluents. Sulfate salts of these metals (purity greater than 99.9%), supplied by the industrial partner, were dissolved at a concentration of 200 mg/L (stock solution) in demineralized water. Monocontaminated solutions of the desired concentration were obtained by simple dilution. Similarly, from these solutions, water polycontaminated with 4 metals (Cu, Zn, Mn and Ni) was prepared at a concentration of 25 mg/L for each metal. This total concentration of 100 mg/L was selected as it is representative of the total metal load found in the industrial partner’s water. The calcium and sodium salts were in chloride form and were also supplied by the industrial partner (purity greater than 99.9%). The exact concentration of each initial solution was measured by inductively coupled plasma atomic emission spectrometry (ICP-AES), as was the pH before and after adsorption.

### 3.3. Experimental Protocol

The analytical technique used to determine the purification performance of wood samples is the so-called batch technique [46], which involves bringing a given mass of material into contact with a volume of solution to be treated under pre-defined experimental conditions. The mixture is then stirred for a certain period of time and filtered. The supernatant is then analyzed. The batch method is widely used in the literature and in the industry, as it is simple to implement, it is fast, and it requires standard equipment. It is also reproducible, making it easy to model results. The procedure used in this study is as follows: 1 g of sample is added to 100 mL of solution to be treated, of known initial concentration and pH; the solution is then stirred at 250 rpm and room temperature for 120 min; at the end of the experiment, the solution is filtered through a 0.45 μm filter; the supernatant is recovered and analyzed by ICP; the pH of the final solution is also systematically measured. This batch protocol is similar to that used by the industrial partner in the surface treatment sector. Several parameters were studied in order to measure their impact on the purification performance of the materials: the initial copper concentration (from 10 to 200 mg/L), the adsorbent dose (from 0.5 to 1.5 g), the contact time (from 5 to 240 min), the pH (from 2 to 5) and the ionic strength; this was achieved by adding two salts (NaCl and CaCl_2_) at concentrations of 0.1, 0.5 and 1 M. All adsorption experiments were repeated (n = 3–5) under identical conditions.

### 3.4. Chemical Analysis

The cation determination was carried out by ICP-AES spectrometry by the PEA^2^t platform of Chrono-environment (Besançon, France). Based on these analyses, the percentage abatement of the cation concerned was calculated according to Equation (1), where C_i_ and C_f_ represent the initial and final concentrations, respectively, expressed in mg/L. The results were also expressed using the amount of metal adsorbed per gram of adsorbent material (q_t_ in mg/g), given by Equation (2), where V represents the volume of solution (L) and m is the mass of material (g).

(1) $$R=\frac{C_{i}-C_{f}}{C_{i}}*100,$$

(2) $$q_{t}=\frac{V(C_{i}-C_{f})}{m}.$$

### 3.5. Modeling

The experimental results for kinetics and adsorption, in a ternary adsorbent/adsorbent/water system, can be modeled by simple, empirical, two-, three- or n-dimensional equations [46,47,48,49,50,51,52,53]. For kinetic results, the models of Lagergren (Equation (3)) [54], Ho and McKay (Equation (4)) [55], Weber and Morris (Equation (5)) [56], and Elovich (Equation (6)) [57] were chosen, and for adsorption data, the adsorption isotherms of Langmuir (Equation (7)) [58], Freundlich (Equation (8)) [59], and Temkin (Equation (9)) [60] were selected. The graphical representation of the various linear equations was used to calculate the maximum quantities of adsorbed copper and the model constants and parameters. The validity of a model was expressed by the value of the regression coefficient of the line and by the accuracy of the calculated parameters with those obtained by experiment [46,47,48,49,50,51,52,53].

(3) $$\ln\;(q_{e}-q_{t})=\ln\;q_{e}-k_{1}t,$$

(4) $$\frac{t}{q_{t}}=\frac{1}{k_{2}{q_{e}}^{2}}+\frac{1}{q_{e}}t,$$

(5) $$q_{t}=k_{p}t^{1/2}+C,$$

(6) $$q_{t}=\frac{ln(\alpha \beta )}{\beta }+\frac{1}{\beta }lnt,$$

(7) $$\frac{C_{e}}{q_{e}}=\frac{1}{K\;q_{m}}+\frac{C_{e}}{q_{m}},$$

(8) $$\ln\;(q_{e})=\ln\;(K_{F})+\frac{1}{n_{F}},$$

(9) $$q_{e}=B\;\ln\;(K_{T})+B\;\ln\;(C_{e}).$$

### 3.6. Microscopic and Spectroscopic Characterization of Materials

Scanning electron microscopy (SEM) analyses were carried out after metallization of the samples by carbon deposition. A field-effect scanning electron microscope (JEOL-JSM_IT500HR, Tokyo, Japan) was used simultaneously for qualitative energy-dispersive X-ray microanalysis (EDS, Brucker, Karlsruhe, Germany). SEM-EDS analysis enables high-resolution image acquisition and chemical analysis of the samples. Images were acquired using secondary electrons at an accelerating voltage of 10 kV and a working distance of 5 mm.

## 4. Conclusions

The results of this study highlighted the value of using a material derived from chemically activated maritime pine fibers to capture copper present in both monocontaminated and polycontaminated metal solutions, whichever concentrations considered. This co-product of the wood industry is interesting not only for its adsorption properties, but also because it is a renewable, abundant and cheap resource. In addition, the proposed chemical modification is relatively simple to implement, free of solvent or other harmful chemicals. Abatements measured for sodium carbonate-activated pine are always higher than those measured for washed pine, proving the efficiency of the activation. These eliminations are high, the reduction being up to 100%. The adsorption process can be considered rapid, as most of the copper was adsorbed within the first 10 min of exposure. Investigating modeling possibilities showed some limitations. Indeed, Weber and Morris and Elovich models showed poor possibilities to describe all the kinetic data for copper adsorption on fibres. This may prove that mechanism is far more complex than simple physisorption, chemisorption and/or diffusion. Lower pH levels were beneficial for performances, whereas a drop in the purification performance of materials with the increase in salt concentration was measured. The proposed material could thus find a new outlet for selective metal recovery. The next step will be to extrapolate these results to other effluents (rinses and baths) and discharges in order to evaluate the impact of the chemical variability of these waters (due to industrial processes) on the purification performance of activated pine fibers. As a consequence, semi-industrial tests on larger volumes are planned. Further spectroscopic characterization of materials, in particular by Raman and XPS measurements as suggested by one of the three reviewers, before and after adsorption, will validate the hypotheses about adsorption mechanisms. Finally, we are currently studying the recovery of metal-laden materials by combustion and their recovery in jewelry (confidential results).

## Figures and Tables

**Table 1:** Theoretical parameters for the kinetic models of Lagergren, Ho and McKay, Weber and Morris, and Elovich (units: C_i_ in mg/L; q_e,exp_ et q_e,cal_ in mg/g; k_1_ in min^−1^; k_2_ in g/mg min et K_p_ in mg/g min^1/2^; α in g/mg et β en mg/g).

			Lagergren			Ho et McKay			Weber et Morris			Elovich		
	C_i_	q_e,exp_	q_e,cal_	k_1_	R^2^	q_e,cal_	k_2_	R^2^	K_p_	C	R^2^	α	β	R^2^
**Pin-W**	25	1.60	0.19	0.014	0.3087	1.59	0.303	0.9980	0.006	1.48	0.1448	6 × 10^35^	58	0.0632
	50	2.06	0.28	0.018	0.3261	2.53	0.250	0.9996	0.013	2.33	0.3825	7 × 10^22^	24	0.2835
**Pin-C**	25	2.53	0.69	0.020	0.7598	2.08	0.122	0.9997	0.052	1.38	0.6661	4 × 10^1^	5	0.8622
	50	4.26	0.101	0.020	0.6089	4.35	0.073	0.9994	0.058	3.47	0.6495	3 × 10^6^	5	0.6049

**Table 2:** Theoretical parameters calculated from the Langmuir, Freundlich and Temkin equations (units: q_m_ in mg/g; K in L/mg; K_F_ in mg^1-(1/n)^ L^1/n^/g; K_T_ in L/g; B in J/mol).

	Langmuir			Freundlich			Temkin		
	q_m_	K	R^2^	1/n_F_	K_F_	R^2^	B	K_T_	R^2^
**Pin-W**	2.2	1.01	0.9813	0.26	0.70	0.8621	0.30	7.9	0.5659
**Pin-C**	3.9	0.10	0.8681	0.31	1.76	0.6002	0.79	27.9	0.4377

**Table 3:** Chemical composition of raw (Pin-R), washed (Pin-W), and activated (Pin-C) pine materials.

	Pin-R	Pin-W	Pin-C
**Klason lignin (%)**	30.7	25.4	26.5
**Lignin soluble (%)**	0.3	0.3	0.3
**Total lignin (%)**	31.0	25.8	26.7
**Extractive soluble water (%)**	4.8	4.4	0.9
**Acetone soluble extractives (%)**	1.7	1.6	0.1
**Total mining and quarrying (%)**	6.5	6.0	1.0
**Total cellulose (%)**	41.9	41.9	48.2
**Total hemicelluloses (%)**	20.0	18.6	21.1
**Ashes at 550 °C (%)**	0.	0.4	0.6

## Data Availability

Not applicable.
